# Common Arterial Trunk in a 3-Day-Old Alpaca Cria

**DOI:** 10.1155/2016/4609126

**Published:** 2016-11-09

**Authors:** Tsumugi Anne Kurosawa, Tamilselvam Gunasekaran, Robert Sanders, Elizabeth Carr

**Affiliations:** ^1^Royal Veterinary College, Clinical Science and Services, Royal Veterinary College, Hawkshead Ln, Hatfield AL9 7TA, UK; ^2^BluePearl Veterinary Partners, 4126 Packard Road, Ann Arbor, MI 48187, USA; ^3^Veterinary Medical Center, Small Animal Clinical Sciences, College of Veterinary Medicine, Michigan State University, 736 Wilson Road, East Lansing, MI 48187, USA; ^4^Veterinary Medical Center, Large Animal Clinical Sciences, College of Veterinary Medicine, Michigan State University, 736 Wilson Road, East Lansing, MI, USA

## Abstract

A 3-day-old alpaca cria presented for progressive weakness and dyspnea since birth. Complete bloodwork, thoracic radiographs, and endoscopic examination of the nasal passages and distal trachea revealed no significant findings. Echocardiogram and contrast study revealed a single artery overriding a large ventricular septal defect (VSD). A small atrial septal defect or patent foramen ovale was also noted. Color flow Doppler and an agitated saline contrast study revealed bidirectional but primarily right to left flow through the VSD and bidirectional shunting through the atrial defect. Differential diagnosis based on echocardiographic findings included common arterial trunk, Tetralogy of Fallot, and pulmonary atresia with a VSD. Postmortem examination revealed a large common arterial trunk with a quadricuspid valve overriding a VSD. Additionally, defect in the atrial septum was determined to be a patent foramen ovale. A single pulmonary trunk arose from the common arterial trunk and bifurcated to the left and right pulmonary artery, consistent with a Collet and Edwards' type I common arterial trunk with aortic predominance. Although uncommon, congenital cardiac defects should be considered in animals presenting with clinical signs of hypoxemia, dyspnea, or failure to thrive.

## 1. Introduction

South American camelids have been reported to have a predisposition to be born with complex congenital heart diseases and it has been hypothesized that this may be associated with the relatively small genetic pool available for breeding outside South America [1]. As such it is important to be aware of the clinical presentation of congenital heart disease in llamas and alpacas. This case report describes the clinical presentation and the physical examination findings of a young cria with complex congenital heart disease. The diagnostic methods used to identify are described and a discussion of the embryologic origins is presented.

## 2. Case Presentation

**Figure 1:**
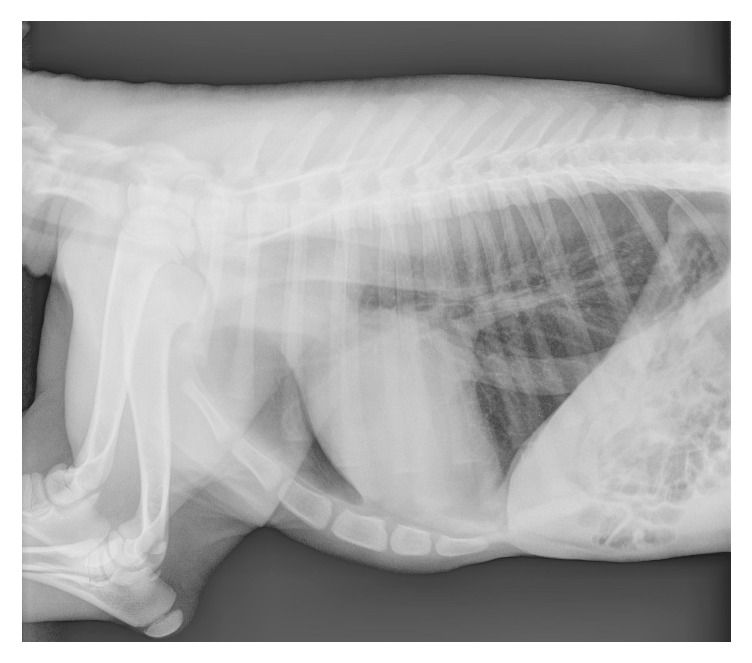
Thoracic radiograph: left lateral thoracic view of the skeletally immature alpaca cria demonstrating mild loss of the cranial waist, distention of the caudal vena cava, and a mild diffuse unstructured interstitial pattern in the lungs warranting cardiac evaluation. A reduced cervical tracheal diameter is also evident.

A 3-day-old, 9.5 kg female alpaca cria was presented for progressive weakness and dyspnea of a few hours' duration. The cria had an uneventful birth, stood, nursed, and passed urine and feces normally, but was less active than normal. The cria nursed regularly but only for very short periods. On presentation, the patient was tachycardic (heart rate 148) and appeared to be dyspneic (respiratory rate 28) with cyanosis of the oral mucous membranes and had a capillary refill time of 3 seconds. Cardiac auscultation revealed no significant abnormalities (excluding the tachycardia) and pulse pressure was considered normal. During examination, the cria intermittently lowered its head, became ataxic, and collapsed. These episodes were precipitated by handling or when nursing. After a few moments the cria sat sternal and then stood, appearing normal. Behavior and neurologic exam between episodes were normal. Differential diagnosis included septicemia, pneumonia, choanal atresia, meningitis, persistent fetal circulation, and cardiac abnormalities. No significant abnormalities were noted on complete blood cell count and blood chemistry. Standard lateral thoracic radiographs (evaluated by a board certified veterinary radiologist) revealed slight loss of cranial cardiac waist, distention of the caudal vena cava, and a mild diffuse interstitial lung pattern (Figure 1) without significant evidence of pulmonary venous congestion or overcirculation. A dorsoventral or ventrodorsal view may have provided additional information regarding the heart, but it is not routinely obtained in an unsedated or unanesthetized cria [1]. An attempt was made to perform upper airway endoscopy to assess for suspected choanal atresia; however, the procedure was aborted as the cria became progressively more distressed. The cria was subsequently anesthetized and placed on 100% oxygen. An endoscopic examination of the nasal passages and distal trachea revealed no significant abnormalities. An arterial blood gas was performed while on oxygen supplementation which revealed a marked hypoxemia (PaO_2_ 19 mmHg, PaCO_2_ 29.6 mmHg, and SaO_2_% 31.7) making cardiac disease with right to left shunting of blood more likely. As such, with no evidence of respiratory diseases or septicemia as the cause of the clinical signs, a congenital cardiac malformation was highly suspected and a cardiac evaluation was performed.

**Figure 2:**
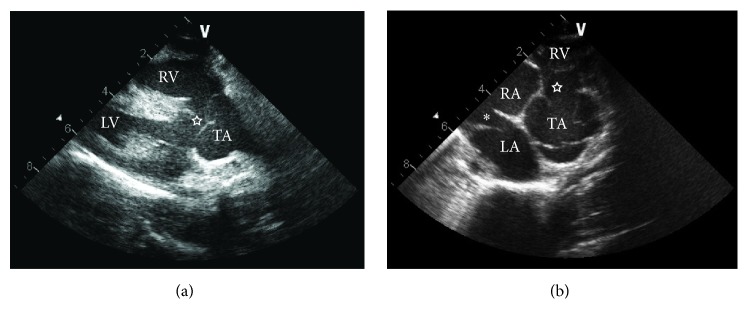
Two-dimensional echocardiographic images. (a) Right parasternal long axis view. Note the truncus arteriosus overriding the ventricular septal defect (star). (b) Right parasternal short axis view at the level of the heart base. Note the ventricular septal defect (star), atrial septal defect (asterisk), and lack of an obvious right ventricular outflow tract. LA, left atrium; RA, right atrium; RV, right ventricle; TA, truncus arteriosus.

**Figure 3:**
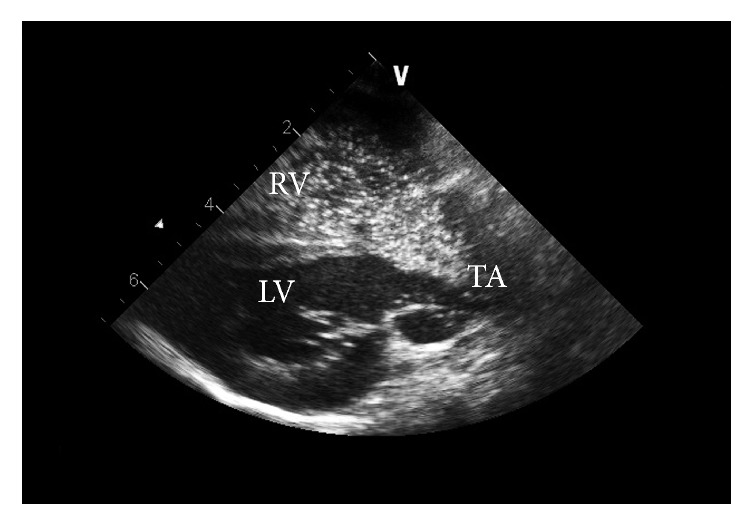
Two-dimensional echocardiographic images of agitated saline contrast study. Two-dimensional echocardiographic right parasternal image of an agitated saline contrast study demonstrating primarily right to left flow of bubbles across the large ventricular septal defect. LV, left ventricle; RV, right ventricle; TA, truncus arteriosus.

Two-dimensional (2D) echocardiography, color flow, and spectral Doppler examinations were performed under general anesthesia with an ultrasound unit (Vivid 7, General Electric Medical System, Waukesha, WI, USA) equipped with 1.5–3.6, 2.2–5, and 4.4–10 MHz phased-array transducers. Two-dimensional images revealed severe dilation of the right atrium and ventricle. Thickening of the right ventricle free wall was also identified. No significant dilation of the left atrium or thickening of the left ventricle was noted. The interventricular septum (IVS) was flattened and there was paradoxical motion of the IVS. At the base of the IVS a large ventricular septal defect (VSD) was detected (Figure 2(a)). Additionally, a patent foramen ovale (PFO) was noted in the atrial septum (Figure 2(b)) and a single large artery overriding the VSD was also identified. The right ventricular outflow tract, origins of the pulmonary arteries, and a patent ductus arteriosus could not be visualized during the echocardiographic examination. Systolic function appeared normal as estimated by 27% fractional shortening (normal 32.8 ± 7.6) [1]. Color flow Doppler evaluation revealed bidirectional but primarily right to left shunting across both the defect in the atrial septum and VSD. Mild regurgitation across mitral, tricuspid, and the valve of the single large artery was noted. A contrast study was performed by injecting agitated heparinized saline into the external jugular vein while viewing the heart from the right parasternal view. Presence of bubbles from the right heart crossing the VSD into the left heart and main artery during systole confirmed the presence of a right to left shunting VSD (Figure 3; Supplementary Information: Video 1 and Video 2 in Supplementary Material available online at http://dx.doi.org/10.1155/2016/4609126). Differential diagnosis based on the echocardiographic findings included Tetralogy of Fallot, severe pulmonic stenosis or pulmonary atresia with a VSD, and common arterial trunk (CAT). Surgical implantation of a vascular shunt and open-heart surgical correction of the malformation was discussed with the owners but due to the poor prognosis and limited treatment options, the owner elected humane euthanasia and postmortem examination.

On macroscopic examination, there was evidence of both right sided (liver congestion and pleural, pericardial, and peritoneal effusion) and left sided (marked pulmonary edema) congestive heart failure. Examination of the heart revealed marked dilation of the right atrium, a small PFO, a large VSD, and a single large vessel overriding the IVS. The vessel had a mildly thickened quadricuspid valve and appeared to be the only outflow tract for both the right and left ventricles consistent with a CAT. A separate pulmonary artery originating from the right ventricular outflow tract could not be identified despite careful dissection. However, a single pulmonary trunk arose from the common trunk prior to the arch and branched to the right and left pulmonary arteries. A patent ductus arteriosus was not identified. Coronary artery structure appeared to be normal. Histological examination identified the presence of pulmonary edema and hepatic congestion consistent with left and right sided congestive heart failure. There was an incidental finding of a cerebellar pseudocyst. Based on the postmortem examination, a diagnosis of a type I CAT (according to Collett and Edwards' classification) and PFO was made.

## 3. Discussion

Common arterial trunk is described as the presence of a single vessel with a semilunar valve, originating from the base of the heart serving as an outlet for both ventricles supplying blood to the systemic, pulmonary, and coronary circulations together with a VSD [2]. In people, CAT is uncommon representing 1-2% of all congenital heart defects with no gender predilection [3, 4]. There have been various associated cardiac anomalies with a right aortic arch (28%) and interrupted aortic arch (18%) as the most frequently reported concurrent defects [4, 5]. In veterinary patients, CAT is rare with a few reported cases in a llama, cats, dogs, cattle, monkey, and a horse [6–14]. The incidence of congenital cardiac diseases in alpacas is currently unknown. However, in a large retrospective of 663 llamas, VSD was the most common congenital cardiac abnormality reported (14/24 cases) [15]. Reports of cardiac defects in camelid species include VSD, patent ductus arteriosus, transposition of the great vessels, persistent right aortic arch, pulmonary atresia with a VSD, and CAT in a llama [1, 6, 15–17]. To the best of authors' knowledge, this is the first report detailing echocardiographic findings of a CAT and PFO in an alpaca. It is not uncommon for the foramen ovale to remain patent for up to 2 weeks in a llama cria and once the left atrial pressure exceeds the right atrial pressure, the embryologic structure closes [15].

Common arterial trunk is widely described as a conotruncal malformation occurring during fetal development. This categorization arose mainly from the use of two outflow tract components: “conus” and “truncus” by the early investigators to classify congenital outflow tract abnormalities [18]. However, recent studies using electron microspy and episcopy on human and mice embryos recognized three outflow tract components, namely, the proximal, intermediate, and distal components in the developing primary heart tube [19]. With ongoing contributions of nonmyocardial tissues from the second heart field and from neural crest cells these outflow tract components transform to form intrapericardial trunks, arterial roots, and subvalvular ventricular outflow tracts, respectively, in the postnatal heart. During development, the common lumen of the distal outflow tract is initially divided into separate intrapericardial arteries by the aortopulmonary septum derived from the dorsal wall of the aortic arch [20]. This is followed by the progressive fusion of the outflow tract cushions that spiral through the intermediate and proximal components. But these septal structures disappear later as the intrapericardial aorta and pulmonary trunks form their own discrete walls. The proximal cushions then fuse with the muscular crest of the right ventricle to form the septal component of the subpulmonary infundibulum [21]. In the left ventricle the posterior wall of the outflow tract is formed by the fibrous extension of the aortic and mitral valve leaflets followed by the closure of the interventricular communications by formation of membranous septum [20].

In normal fetal development the spiral septum separates the common trunk into the pulmonary trunk and proximal ascending aorta, allowing proper delivery of deoxygenated and oxygenated blood, respectively. Failure of fusion of the outflow tract cushions results in formation of a single large trunk with a common ventriculoarterial junction and a single semilunar valve, overriding a VSD [22]. The common trunk supplies a mixture of oxygenated and deoxygenated blood into the systemic, pulmonary, and coronary circulations [2]. The VSD associated with CAT is typically large, extending from the truncal valve to the pars membranacea septi [3]. There is usually a fibrous tissue connecting the septal leaflet of the mitral valve to the noncoronary leaflet of the truncal valve which distinguishes CAT from a form of transposition of the great arteries [3]. The CAT more often originates from both either ventricles or the right ventricle and less frequently the left ventricle [4, 5]. The truncal valve is most commonly tricuspid but can be bicuspid, quadricuspid, or pentacuspid [2]. The valves can have marked changes such as abnormal thickening that can lead to truncal insufficiency or stenosis [2].

Common arterial trunk is often categorized according to a classification system based on the origins of the pulmonary arteries [23]. In type I, the most common type in humans, a single pulmonary trunk arises from the CAT, which subsequently bifurcates into the right and left pulmonary arteries [4]. In types II and III, separate right and left pulmonary arteries arise directly from the CAT [23]. In type II the arteries originate from the dorsal wall of the CAT while in type III they are more laterally oriented [23]. The pulmonary artery is absent in type IV (absence of the 6th aortic arches) and blood supply to the lungs is via the bronchial arteries and is now considered to be pulmonary atresia with a VSD [4, 23]. A second classification system was later described primarily based on the presence (type A) or absence (type B) of a VSD and development of the aorticopulmonary septum [2]. Most cases of type B were actually aortopulmonary window defects, and as such type B classification is no longer used [4]. Type A is subdivided into 4 categories with type A1 corresponding to Collett and Edwards type 1 and type A2 to Collett and Edwards type II and type III. Type A3 describes an absence of one pulmonary artery with a presence of a ductus arteriosus or collateral vessels to provide blood flow to the lungs [3]. Finally, type A4 includes hypoplasia, coarctation, and atresia or absence of the aortic arch [3]. Recently, a simplified categorization that classifies CAT based on the presence of systemic or pulmonary predominance was proposed [24, 25]. In pulmonary dominance, the common trunk trifurcates into right and left branch pulmonary arteries and ductal continuation to descending aorta. The ascending aorta emerges from common trunk as a side branch and is typically hypoplastic [24]. In aortic dominance, the common trunk resembles the ascending aorta continuing to have normal aortic arch. The pulmonary arteries originate from the left posterior aspect of the trunk [24]. This simplified approach helps in surgical risk stratification since the number one risk factor defining surgical mortality is the presence of hypoplastic or interrupted aortic arch [25]. For the cria of this report, a single pulmonary trunk arose from the common trunk before bifurcating into right and left pulmonary arteries prior to the aortic arch consistent with the type aortic predominance.

Clinical presentation of veterinary patients with CAT can be variable. As with this case, newborn patients can present with severe clinical signs shortly after birth while others may be asymptomatic or develop clinical signs later in life and go undetected for several years [8, 10]. In humans, CAT is usually detected early in infancy (or in utero), and early intervention can improve long-term survival [26]. Patients usually present with symptoms of congestive heart failure soon after birth and survival to adulthood is rare without correction [27–29]. Accurate echocardiographic diagnosis of CAT is challenging. Differential diagnosis based on echocardiographic findings included CAT, Tetralogy of Fallot, and severe pulmonic stenosis or pulmonary atresia with a VSD. In Tetralogy of Fallot, a large VSD with an overriding vessel is identified, but unlike CAT, a separate pulmonary artery is present. In pulmonary atresia there is an overriding aorta with atresia of the pulmonary artery which can appear very similar to a CAT on echocardiographic examination. Pulmonary circulation in pulmonary atresia can be supplied by a concurrent patent ductus arteriosus or bronchial arteries whereas in CAT, distinct pulmonary arteries arise from the trunk as previously described [4, 29]. As such, a diagnosis of CAT must identify a single arterial vessel giving rise to systemic, pulmonary, and coronary arteries. Cardiac catheterization and angiography or magnetic resonance imaging may be required to obtain an accurate antemortem diagnosis [2]. Contrast studies such as one performed in this case can provide additional information regarding the hemodynamics of the anomaly.

The classification of CAT and origin of the trunk are important factors when evaluating patients before corrective surgery. For example, patients with only one pulmonary artery often develop severe pulmonary vascular disease even with correction during the neonatal period [30]. In people, surgical correction involves separating the pulmonary arteries from the CAT and creating a connection to the right ventricle via a valved conduit and the CAT is then connected to the left ventricle and the VSD is closed [26]. Complete surgical repair early during infancy is preferred as progression of pulmonary vascular obstructive disease can increase mortality postoperatively [2]. In the last 30 years, the improvements in surgical techniques and postoperative management have contributed to a favorable prognosis in people after surgical repair of CAT with over 90% survival for one year postoperatively [26]. Without surgical repair, 80% of human patients do not live past one year of age [29]. Factors impacting long-term survival include age at diagnosis, conduit stenosis, truncal valve insufficiency, truncal valve stenosis, myocardial failure, pulmonary hypertension, and concurrent interrupted aortic arch [26]. The prognosis in veterinary patients is currently unknown as reported cases are lacking but is presumed to be poor. At this time surgical repair has not yet been described in the veterinary literature.

This case describes an unusual complex cardiac anomaly in a camelid species. The alpaca cria described in this report presented with severe clinical signs that were associated with right to left shunting of blood due to a type I CAT with PFO. The radiographic evaluation of the lung fields did not match the finding noted on necropsy. This may have been due to progression of the patient's condition to severe respiratory distress after the radiographs were taken. To the best of authors' knowledge, this is the first case report detailing the echocardiographic findings associated with CAT in an alpaca cria. Although uncommon, congenital cardiac defects should be considered in animals presenting with clinical signs of hypoxemia, dyspnea, or failure to thrive.

## Supplementary Material

Video 1: Agitated saline contrast study. A cineloop of a two-dimensional echocardiographic right parasternal long axis view and color Doppler demonstrating the bidirectional flow across the large ventricular septal defect (VSD) and aortic regurgitation. Note the right to left flow across the VSD during systole and left to right flow across the VSD during diastole.
Video 2: Agitated saline contrast study. A cineloop of a two-dimensional echocardiographic right parasternal view including the truncus arteriosus after injection of agitated heparinized blood. Note the primarily right to left direction of bubble flow across the large ventricular septal defect.
